# The Study on *Culicoides*: The Environment They Live in and Trypanosomatids They Coexist

**DOI:** 10.3390/insects16080770

**Published:** 2025-07-27

**Authors:** Margarita Kazak, Kristina Valavičiūtė-Pocienė, Rasa Bernotienė

**Affiliations:** State Scientific Research Institute Nature Research Centre, Akademijos Str. 2, LT-08412 Vilnius, Lithuania

**Keywords:** biting midges, Ceratopogonidae, dixenous, monoxenous, parasites, seasonality, Trypanosomatidae

## Abstract

Information on *Culicoides* transmitted parasites, especially trypanosomatids, infecting animals and insects, is scarce. Our goal was to clarify the seasonal patterns of both *Culicoides* and trypanosomatids detected in these insects and the relationships between *Culicoides* abundance and meteorological parameters. UV light traps were used to collect biting midges in four study sites in 2022–2023; collected *Culicoides* females were dissected and analyzed using microscopy and PCR-based methods. Out of 1631 parous *Culicoides* females belonging to 14 different species, 6.5% were found to be infected with trypanosomatids (5.0% with at least three *Trypanosoma* species and 1.5% with monoxenous parasites). The highest *Culicoides* abundance was detected in June. The prevalence of trypanosomatids in biting midges increased during the summer (5.3% in June, 8.8% in July, and 11.2% in August). Temperature was recorded to be a presiding environmental gradient structuring *Culicoides* species composition, while wind speed and precipitation explained little of the variation. Our results indicate that both avian and mammalian trypanosomes can be found in these insects, although further research is needed to better understand the development of these parasites in biting midges and *Culicoides* vectorial capacity.

## Simple Summary

Biting midges are among the smallest blood-sucking insects that transmit various pathogens to humans and animals. Due to the climate crisis, these insects can move into new areas, introducing new pathogens that were not present there before, indicating the importance of a seasonal surveillance of these insects. Although there have been some studies on *Culicoides* seasonality and their relationships to meteorological parameters, there is much less information on the seasonality of the parasites transmitted by these insects, especially trypanosomatids infecting wild and domestic animals and insects. During a two-year study, we tested 1631 biting midge females belonging to 14 species using microscopy and PCR-based methods for the presence of trypanosomatids. The highest *Culicoides* abundance was detected in June, but the highest prevalence of trypanosomatids in these insects was detected in August. Our results show that the activity of some *Culicoides* species is related to air temperature. At least two species of monoxenous trypanosomatids and three *Trypanosoma* species were detected in biting midges, and the morphometrical analysis of *Trypanosoma* indicates the importance of using both microscopy and PCR-based analysis for parasite identification.

## 1. Introduction

*Culicoides* Latreille, 1809 (Diptera: Ceratopogonidae) biting midges are well-known worldwide, ranging from the tropics to the subtropics, tundra, and temperate regions (except for Antarctica and New Zealand) [1,2], and while being among the smallest Diptera vectors, they are capable of transmitting arboviruses, bacteria, protozoa, and helminth parasites to humans and animals all around the world [3]. The latest studies have noted the importance of these insects as vectors of avian parasites (hemosporidian parasites (Haemosporida) of the genus *Haemoproteus* Kruse, 1890 and trypanosomatids (Trypanosomatida)) [4,5,6,7]. Experimental studies on *Culicoides* capability to transmit various avian pathogens mostly revolve around a few species [4,5,8,9,10,11], as the biological and ecological characteristics of these insects make their cultivation in laboratories complicated and require additional effort [12,13].

In a changing world with climate crisis and increasing mobility, the importance of research on the transmission of human and animal parasites is becoming evident. Due to a changing climate, the geographical ranges of insects are expanding, and at the same time, the ranges of the parasites transmitted by insect vectors are expanding along [14,15]. Although biting midges are weak fliers—they mostly stay within about 2 km around their breeding sites [2,16,17], although Elbers et al. indicated that some species might travel up to 5 km [1]. With the help of the wind, these insects can be moved thousands of kilometers, especially over the sea, introducing pathogens into new areas in this way [18]. *Culicoides* biting midges might possess the potential to expand their geographic ranges and possibly contribute to the spread of vector-borne pathogens due to climate crisis. *Culicoides* adults may be present throughout the whole warm season in temperate zones—from early spring until autumn depending on the species [19]. While there is sufficient knowledge about the seasonal variations of several *Culicoides* species [20,21], there is much less information regarding the seasonality of parasites transmitted by these insects, especially regarding trypanosomatids infecting wild and domestic animals and insects.

Trypanosomatids (Kinetoplastea: Trypanosomatidae) include both obligate monoxenous (living in one host—invertebrate) and dixenous (living in two—invertebrate and vertebrate hosts) parasites [22]. Although there is not enough data on the impact of trypanosomatids on their hosts, some of them may be pathogenic and affect the development and behavior of heavily infected individuals [23,24], and in some cases, even lead to increased host mortality [25]. Genus *Trypanosoma* Gruby, 1843 is one of the best studied in the family Trypanosomatidae, however, most investigations have focused on species pathogenic to human and domestic animals [26,27], and much less research has been conducted on wild bird-infecting species, especially ones that are detected in wild-caught vectors. Moreover, there is no information about the seasonality of the prevalence of these pathogens (both monoxenous and dixenous) in insects.

The transmission of vector-borne diseases is influenced by several factors including the free accessibility of infected hosts, the activity of competent vectors, and optimal meteorological conditions. In the absence of continuous entomological surveillance in disease endemic areas, it is essential for health authorities to have information on potential vector distribution and flight activity so that potential transmission risk areas and periods can be quickly identified during an outbreak [28,29]. Although there is no data on what parameters are the most important for avian trypanosome development in invertebrate hosts, some studies on human-infecting *Trypanosoma* show that lower temperatures might impact the metacyclogenesis rate of *Trypanosoma cruzi* Chagas, 1909 in Triatominae bugs [30], and the average epimastigote population density and average blood consumption were recorded to significantly differ between insects stored at 28 °C that could and could not maintain differentiation [31]. A study on temperature effect on avian blood hematozoan parasites showed that higher altitudes with low temperatures may lead to the reduced development of hematozoa and the lower abundance of parasite vectors, hence the prevalence of the parasites they transmit. More importantly, the study showed that the rise in temperatures can lead to higher parasite prevalence in avian hosts [32]. Such studies emphasize the complexity of host–parasite interactions and the impact that the changing environment may have on them. Information on how environmental changes impact trypanosomatids in *Culicoides* is still scarce and requires more research.

Molecular research and microscopic examination are widely used for parasite research in both the digestive tract and vertebrate blood and tissue of insects [33,34,35]. Each method has its advantages: although requiring more time, experience, and diligence, microscopic examination is useful for investigating the low parasitemia and mixed infections that often occur in the wild, while PCR-based analysis provide valuable data (DNA sequence information) that can be used for the investigation of population genetics, phylogenetics, epidemiology, and other studies, however, PCR-based methods are more expensive and usually do not detect mixed infections of trypanosomatids as well as other parasites [33,34,36]. Nevertheless, molecular analysis can supplement results obtained by microscopy [37], especially for species that are morphologically undistinguishable [38].

We combined both microscopy and PCR-based methods to investigate wild-caught *Culicoides* biting midges and their trypanosomatid parasites. Our aim was to understand their seasonality and how meteorological parameters such as wind speed, precipitation, and air temperature can influence both biting midge activity and their capability to be infected with trypanosomatids.

## 2. Materials and Methods

### 2.1. Insect Trapping

**Figure 1:**
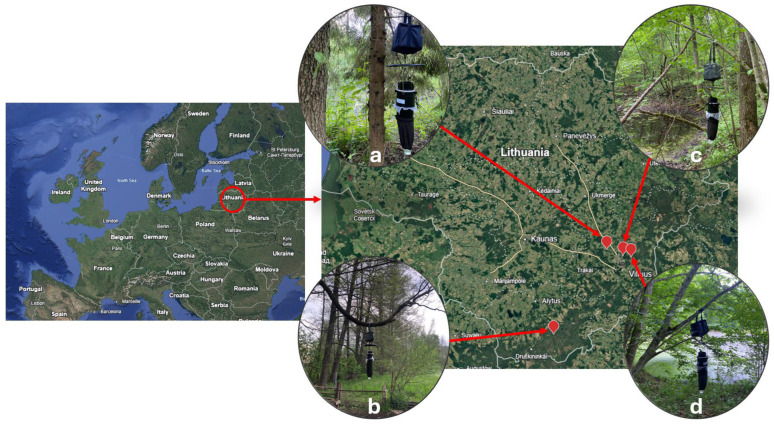
Map indicating the trapping sites of this study: near an artificial dich in the forest, Brinkiškės (**a**); over sheep grazing area near Puvočiai (**b**); next to a naturally occurring puddle in Verkiai (**c**); and next to an artificial stagnant water pond in the Botanical Garden (**d**).

Biting midge collection was implemented every two weeks during the warm season, from May to September in 2022–2023 at three study sites in the southern part of Lithuania (Brinkiškės village (54°79′88.4″ N, 25°06′03.7″ E) (further—Brinkiškės), Verkiai Regional Park (54°45′00″ N, 25°17′00″ E) (further—Verkiai), and Vilnius University Botanical Garden (54°44′12.5″ N, 25°24′16.4″ E) (further—Botanical Garden)). In June of 2022 and 2023, the material was also collected in Puvočiai village (54°06′52.2″ N, 24°18′17.6″E) (further—Puvočiai), south Lithuania (Figure 1). All study sites were characterized by the presence of water bodies nearby and a dense covering of tree foliage. They represented four different environments: Puvočiai represents rural areas with a high density of domesticated animals (cattle, sheep, cats and dogs, chicken); Brinkiškės represents a mixed forest area further from the city with a high density of wild cervids, small mammals, and birds (personal observation); the Botanical Garden is characterized by mixed forest on the city outskirts affected by human activity with a high density of birds and several artificial ponds nearby; and Verkiai represents a mixed forest area on the city outskirts with a high density of birds.

BG-Pro UV-light traps (Biogents, Regensburg, Germany) were used (Figure 1) to collect biting midges. During each trapping session, two traps per site were used; there was always at least a 10 m distance between the traps.

Traps were hung on tree branches at a height of 1.5–2 m; these were turned on 4–5 h before sunset and turned off 1–2 h after sunrise. The material was collected in 100 mL water containers with a drop of liquid soap to break the water surface tension, allowing the insects to sink. Collected material was immediately transported to the laboratory on the same morning for further processing.

### 2.2. Culicoides Identification

Only parous biting midge females were investigated; these were determined based on the burgundy pigment on the abdomen, as described by Dyce in 1969 [39]. Parous females were identified using the interactive identification key for *Culicoides* (Diptera: Ceratopogonidae) [40]. Identification was based on the main morphological biting midge features—wing coloration patterns and head features such as palps and antenna. For this purpose, permanent preparations of the head and wings were prepared for a further examination of biting midges using microscopy: head and wings were placed in a drop of Euparal on a glass slide and covered with a cover slide. The prepared slides were left to dry for approximately a month at room temperature before further processing.

### 2.3. Microscopy of Parasites

After preparation of the wing and head slides, the insect abdomen was removed and placed in a drop of 0.9% saline solution. The gut was extracted using flame-disinfected needles (to avoid DNA contamination); the extracted midgut was crushed on an objective slide to prepare a thin film. Preparations were air-dried, fixed with absolute methanol, and stained with Giemsa as described by [33]. The remnants (thorax and abdomen remnants) were stored in 96% ethanol for PCR-based investigation.

An Eclipse C1-L light microscope (Nikon, Tokyo, Japan) equipped with an Infinity 1 digital camera (Teledyne Lumenera, Waterloo, ON, Canada) and Infinity Analyze imaging software 6.5.6 (Teledyne Lumenera, Waterloo, ON, Canada) was used for the slide examination, illustration preparation, and further morphometrical analysis of trypanosomatids. Morphometrical features used in this study were based on [41,42,43].

### 2.4. PCR-Based Screening and Sequencing

Total DNA was extracted from all dissected parous biting midge remnants using ammonium-acetate DNA precipitation protocol [44]. For trypanosomatid detection, a nested PCR protocol [43,45] for the amplification of the 749 bp length DNA fragment encoding SSU 18S rDNA was used with outer primers TRYP 763/TRYP 1016 and inner primers TRYP 957/TRYP 99 (Table 1). All PCRs were performed in 25 μL total volume (12.5 μL of DreamTaq Master Mix (Thermo Fisher Scientific, Vilnius, Lithuania), 8.5 μL nuclease free water, 1 μL of each primer (10 pmol), and 2 μL of the total genomic DNA template or PCR product in case of the nested PCR). Temperature profiles in all PCRs were the same as in the original protocol’s description: first, PCR started with denaturation at 95 °C (5 min), followed by the first 5 cycles of 95 ° (60 s), 45 °C (30 s), 65 °C (60 s), and then 35 cycles of 95 °C (60 s), 50 °C (30 s), 72 °C (60 s); final extension—65 °C (10 min). For the nested PCR: denaturation at 96 °C (3 min), following 25 cycles of 96 °C (30 s), 58 °C (60 s), 72°C (30 s), with a final extension at 72 °C (7 min). During each amplification run, one positive control (2 μL of *Trypanosoma* sp. infection confirmed by both microscopic examination and PCR) and one negative control (2 μL of ultrapure water) were used. For the evaluation of amplifications, 3 μL of the final PCR products were run on 2% agarose gels.

For insect species confirmation, a PCR with primers LCO 1492 and HCO 2198 (Table 1), which amplifies a fragment of Cytochrome c oxidase subunit 1 (COI) of mtDNA, was run with temperature profiles used as described by [46].

All positive samples were sequenced with corresponding primers using the Big Dye Terminator V3.1 Cycle Sequencing Kit and ABI PRISMTM 3100 capillary sequencing robot (Applied Biosystems, Foster City, CA, USA). Obtained sequences were aligned and analyzed using Geneious Prime^®^2023.0.4 (Dotmatics, Auckland, New Zealand, https://www.geneious.com, accessed on 1 April 2023) software in order to create a consensus sequence. Obtained sequences were assigned to a species using the ‘Basic Local Alignment Search Tool’ (NCBI BLAST, https://blast.ncbi.nlm.nih.gov/Blast.cgi, accessed on 10 January 2023) by comparing the consensus sequence with sequences deposited in GenBank [47]. Identifications were confirmed only for the sequences that presented a similarity of more than 98% with other sequences deposited in the database.

### 2.5. Statistical Analysis

Meteorological parameters, the average of a week (wind speed (m/s), precipitation (mm), and air temperature (°C)), were obtained from the Lithuanian Hydrometeorological Service (www.meteo.lt, accessed on 10 February 2025) from the stations located closest to the study sites. The evaluation of the relationship between the meteorological parameters and *Culicoides* abundance of different species was determined by applying redundancy analysis (RDA) using Brodgar software, version 2.7.5 (Highland Statistics Ltd., Newburgh, UK). Prior to RDA, we conducted a detrended correspondence analysis (DCA) on the species matrix to assess the gradient lengths of the first axis. The resulting axis length was less than 3, indicating that a linear response model was appropriate, thus justifying the use of RDA over canonical correspondence analysis (CCA). Species abundance data were transformed using the Hellinger transformation. The analysis was performed in R (version 4.4.1.) using the “vegan” package [48]. Samples with zero total abundance (i.e., no *Culicoides* caught) were excluded from the analysis to avoid biasing the ordination with structurally empty community data. Material collected in Puvočiai was not included in the RDA analysis and seasonal biting midge activity investigation, as trapping there was conducted for only one month per season. Only the most abundant species (individuals of the species that were detected in more than 5 trappings) were included in the analysis. Statistical significance of the RDA model and its components was tested using a permutation test (999 permutations) implemented in the “vegan” package in R. Significance was assessed for the overall model, individual axes, and each environmental variable. To assess multicollinearity among the environmental variables (wind, precipitation, temperature), variance inflation factors (VIFs) were calculated.

The parasite prevalence (%) was calculated as the proportion of infected biting midges among all the investigated midges. Fisher’s exact test was used to compare the prevalences of *Trypanosoma* parasites between different biting midge species and different years of investigation. Simpson’s diversity index (D) was calculated for each study area using Microsoft Excel to assess the species diversity of *Culicoides* biting midges.

To visualize and compare the variation in each morphological trait across biting midge species and *Trypanosoma* species, boxplots were generated using R. Boxplots were created using the “ggplot2” package in R to display the distribution of each trait across species, with consistent axis scaling applied to allow for a visual comparison between traits.

Statistical differences in trait measurements between groups were assessed using one-way ANOVA. Separate ANOVAs were performed to test differences amongst biting midge species and between *Trypanosoma* species. All analyses with a *p* < 0.05 were considered statistically significant. Post hoc comparisons were performed using Tukey’s HSD test where necessary.

## 3. Results

### 3.1. Seasonal Culicoides Abundance

**Figure 2:**
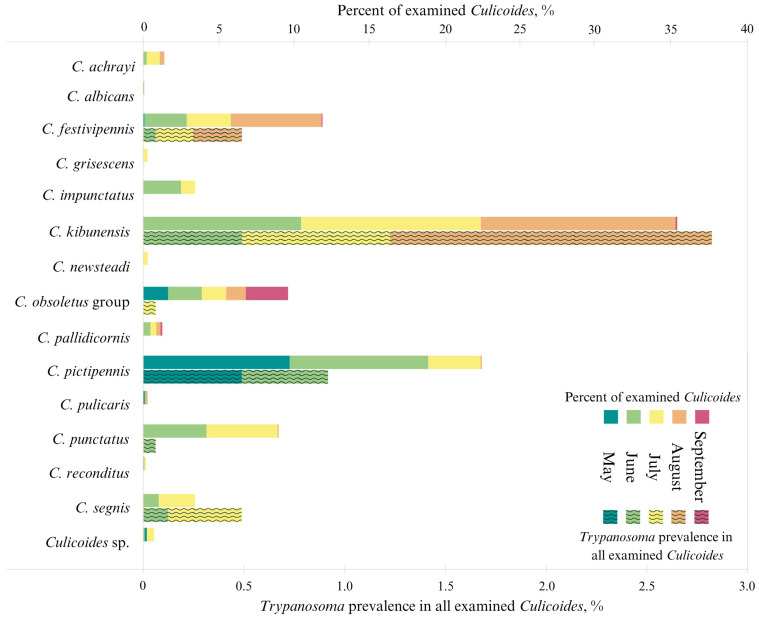
The relative abundance of the investigated *Culicoides* with the prevalence of *Trypanosoma* during May–September of 2022–2023 in Brinkiškės, Verkiai, and the Botanical Garden.

Overall, 9579 biting midges were caught, but only 1631 of them were parous females, which were dissected and investigated. We determined 14 *Culicoides* species (Figure 2). Ten biting midge specimens could not be identified morphologically to the species level due to damaged specimens. The highest abundance of parous *Culicoides* was detected in June (30.8% of all investigated parous biting midge females), while the least number of individuals was caught in September. *Culicoides kibunensis* Tokunaga, 1937 accounted for the vast majority of the collected biting midges (n = 452, 35.5%), following *Culicoides pictipennis* (Staeger, 1839) (n = 287, 22.5%) and *Culicoides festivipennis* Kieffer, 1914 (n = 152, 11.9%); other species accounted for less than 10% (Figure 2). *Culicoides pictipennis*, *C. obsoletus* group, *C. kibunensis*, *Culicoides impunctatus* Goetghebuer, 1920, *C. festivipennis*, *Culicoides punctatus* (Meigen, 1804), and *Culicoides segnis* Campbell and Pelham-Clinton, 1960 prevailed consistently throughout the season (from May to September), while *Culicoides albicans* (Winnertz, 1852), *Culicoides achrayi* Kettle and Lawson, 1955, *Culicoides grisescens* Edwards, 1939, *Culicoides newsteadi* Austen, 1921, *Culicoides pulicaris* (Linnaeus, 1758), *Culicoides reconditus* Campbell and Pelham-Clinton, 1960, and *Culicoides pallidicornis* Kieffer, 1919 only occurred at limited times during the season.

Additionally, in Puvočiai, in June, the *C. obsoletus* group accounted for the majority of collected parous *Culicoides* (n = 114, 31.9%), followed by *C. impunctatus* (n = 101, 28.3%), *C. pictipennis* (n = 95, 26.6%), *C. punctatus* (n = 27, 7.6%), *C. kibunensis* (n = 11, 3.1%), and *C. festivipennis* (n = 4, 1.1%); other species accounted for less than 1%.

*Culicoides albicans* were only caught in June, *C. grisescens* were only caught in July, and *C. reconditus*, *C. segnis*, and *C. newsteadi* were caught in June and July (Figure 2). The differences in the abundance of certain species were determined between different years (e.g., *C. kibunensis* made up 52.1% of all collected biting midges in 2022 and was only 32.1% in 2023; for *C. pictipennis,* these proportions were 5.3% and 38.9%, respectively, and for *C. punctatus*, these were 1.3% and 16.1%, respectively. Fisher’s exact test confirmed the statistical reliability of these differences (*p* < 0.000).

**Figure 3:**
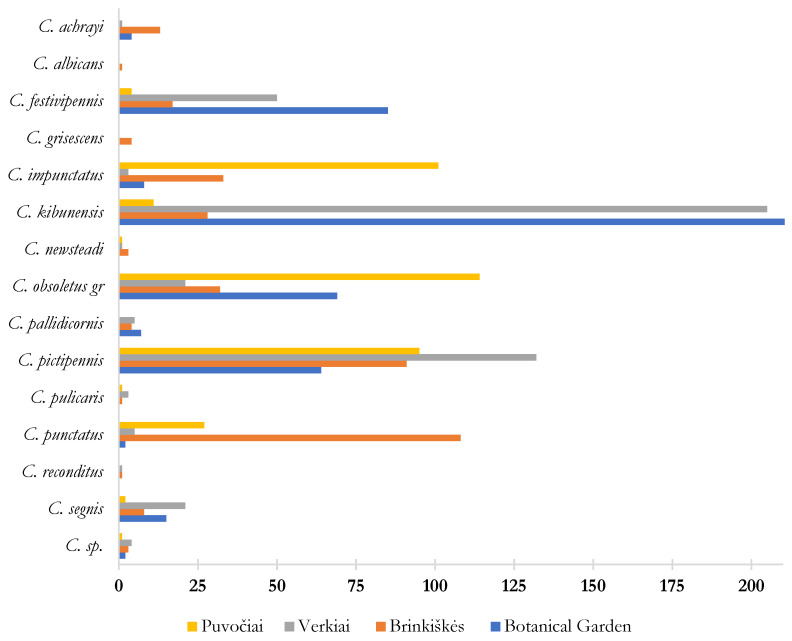
Numbers of collected parous *Culicoides* females at the different study sites from 2022 to 2023.

Different species compositions at different study sites were also observed (Figure 3). The forest area of Brinkiškės could be characterized by the highest diversity of *Culicoides* species—14 (D = 0.81 showing a high degree of diversity) while a smaller number of species and lower diversity were detected in Verkiai (12 species; D = 0.69). In both Puvočiai (D = 0.74, a moderately high degree of diversity) and the Botanical Garden (D = 0.72), nine *Culicoides* species were caught. The highest abundance of parous *C. kibunensis* females was detected at the Botanical Garden (n = 219) and Verkiai (n = 205), whereas at other study sites, the catches were much lower (Figure 3). Similarly, *Culicoides pictipennis* was abundant at all study sites, although the highest abundance was observed in Verkiai (Figure 3). Mammalophilic biting midges—*C. obsoletus* group and *C. impunctatus*—were the most abundant in Puvočiai (Figure 3), and *Culicoides punctatus* was dominant in Brinkiškės (108 individuals collected overall). One specimen of *C. albicans* and four specimens of *C. grisescens* were collected in Brinkiškės and were not present at the other study sites. *Culicoides segnis*, *C. punctatus*, *C. pictipennis*, and *C. obsoletus* group biting midges, *C. kibunensis*, *C. impunctatus*, and *C. festivipennis* were present at all four study sites (Figure 3).

**Figure 4:**
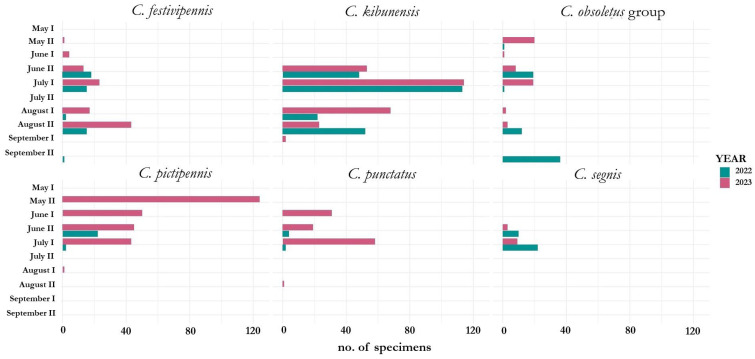
The number of specimens for the six most abundant *Culicoides* species during the season. I represents the first part (from the 1st to 15th day) of the month, and II represents the second part (from the 16th to the last day) of the month.

The number of specimens for the six most abundant *Culicoides* species (*C. festivipennis*, *C. kibunensis*, *C. obsoletus* group, *C. pictipennis*, *C. punctatus*, and *C. segnis*) were analyzed during the seasons over two years (Figure 4).

Females in the *C. festivipennis* and *C. obsoletus* groups were caught throughout the whole warm period—from the second part of May to September (Figure 4), while *C. pictipennis*, *C. punctatus*, and *C. segnis* were more active in the first part of summer. The abundance of the same species differed between the two years of investigation. *Culicoides pictipennis* and *C. punctatus* were caught earlier and more abundantly in 2023 compared with 2022 (Figure 4). The first females of *C. festivipennis* and *C. pictipennis* were caught in May in 2023, with the average air temperature being 14.7 °C at this time, and the first females of the same species were only detected at the end of June in 2022, with the average temperature at this time being 17.5 °C. The first catches of *C. kibunensis* and *C. punctatus* were in June in both 2022 and 2023. *Culicoides segnis* was only recorded from the second part of June until the first part of July (Figure 4). Three species—*C. festivipennis*, *C. kibunensis*, and *C. obsoletus* group—were recorded in September, while *C. punctatus* and *C. pictipennis* were caught until July (2022) and August (2023).

### 3.2. Relationships Between Biting Midge Abundance and Meteorological Parameters

**Figure 5:**
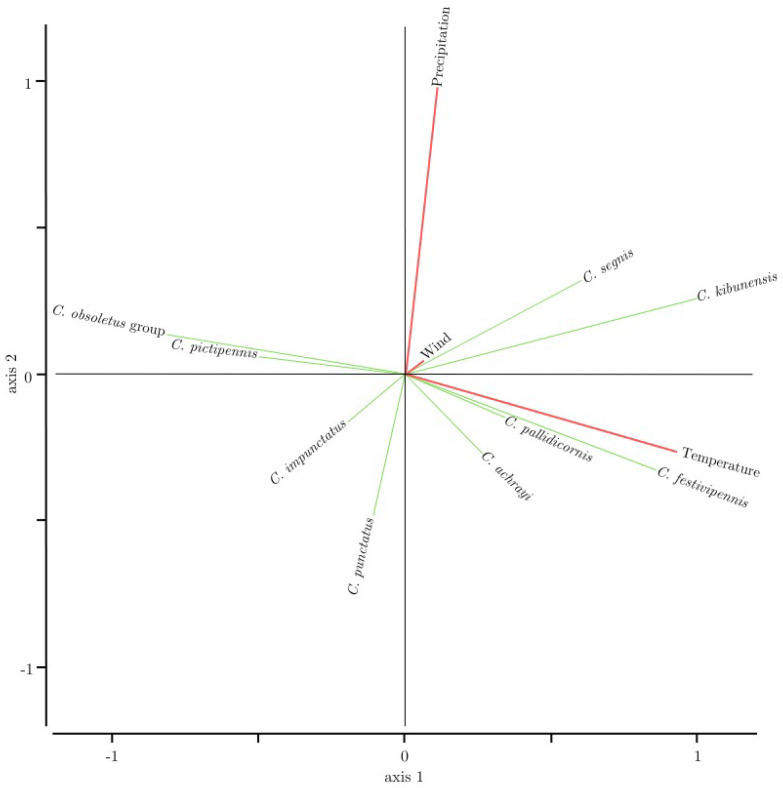
Redundancy analysis revealing the relationships between *Culicoides* abundance of certain species with meteorological parameters (air temperature, precipitation, wind speed). The two-dimensional approximation explained 92.5% of this (76.9% on axis 1 and 16.1% on axis 2).

The RDA biplot shows that temperature and precipitation are the dominant environmental gradients structuring biting midge species composition (Figure 5). All VIF values were below 1.1, indicating very low collinearity and justifying the inclusion of all variables in the RDA model. The overall RDA model was statistically significant (F = 3.92, *p* = 0.001), indicating that the environmental predictors collectively explained a meaningful portion of species variation. Only the first RDA axis (axis1) was significant (*p* = 0.001), suggesting that it captured the primary gradient of the community–environment association. Among the tested variables, only temperature had a significant individual effect on species composition (*p* = 0.001), while wind and precipitation did not show significant contributions (*p* > 0.2). *Culicoides segnis* and *C. kibunensis* were positioned between the temperature and precipitation vectors, suggesting that their abundance may be influenced by both variables, but temperature played the primary role based on permutation tests. *Culicoides festivipennis*, *C. pallidicornis*, and *C. achrayi* were associated more with higher temperatures. Wind speed explained a little of the variation. Some species, such as *C. impunctatus*, *C. obsoletus* group, and *C. pictipennis*, appeared to be weakly associated or negatively associated with the measured environmental variables.

### 3.3. Prevalence of Trypanosomatids in Culicoides

Out of all tested parous females, 6.5% (n = 107) (either PCR, microscopy, or both) were found to be infected with trypanosomatids (1.5% (n = 25) with monoxenous and 5.0% (n = 82) with dixenous) (Table 2 and Figure 2). Both mammalian and avian trypanosomes were detected: *Trypanosoma bennetti* group trypanosomes were the most abundant in *C. kibunensis*, this parasite was also found in *C. obsoletus* group biting midges; *C. pictipennis* and *C. festivipennis*; six *C. segnis* and one *C. kibunensis* individuals were found to be infected with *Trypanosoma avium* Danilewski, 1885 parasites; six biting midges were found to be infected with *T. theileri* group trypanosomes: 1 *C. obsoletus* group female, 1 *C. impuctatus*, 1 *C. punctatus*, and 3 *C. kibunensis*. One *C. segnis* and one *C. festivipennis* were infected with avian trypanosomes that could not be assigned to species (Table 2).

The highest prevalence (n = 4, 0.3%) of monoxenous trypanosomatid *Herpetomonas ztiplika* Podlipaev et al., 2004 was detected in biting midges from the *C. obsoletus* group; this protozoan was also found in *C. pictipennis* and *C. punctatus* (Table 2). *Crithidia brevicula* Frolov and Malysheva, 1989 was detected in *C. pictipennis*, *C. impunctatus*, *C. segnis*, *C. newsteadi*, *C. achrayi*, *C. grisescens*, and *Culicoides* sp. (Table 2).

**Figure 6:**
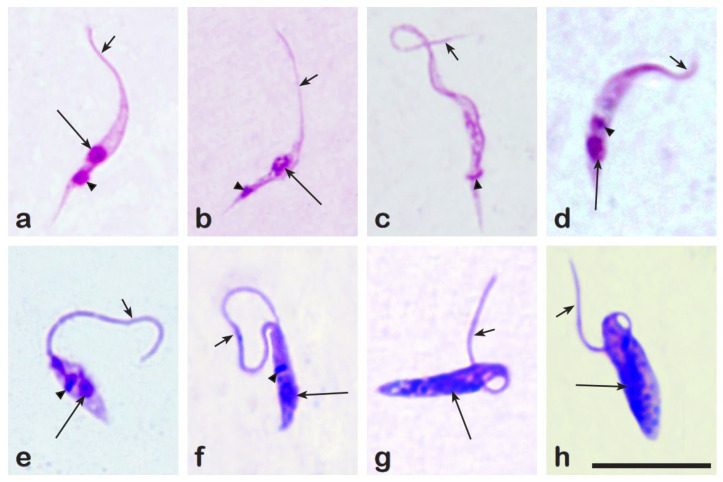
Trypanosomatids in the guts of *Culicoides* biting midges: *Trypanosoma bennetti* group parasites in *C. kibunensis* (**a**) and in *C. pictipennis* (**b**); *Trypanosoma* sp. in *C. festivipennis* (**c**); *T. avium* in *C. segnis* (**d**); *Herpetomonas ztiplika* in *C. punctatus* (**e**) and in *C. pictipennis* (**f**); *Crithidia brevicula* in *C. segnis* (**g**,**h**). Short arrow—flagellum, long arrow—nucleus, triangle arrow—kinetoplast. Scale bar—10 µm.

Out of all the positive for trypanosomatid biting midges, only 54.0% (n = 60) were found to be positive by both PCR and microscopy (Figure 6). Four biting midges (3.6%) were positive only by microscopy and were negative by PCR, and 42.4% (n = 47) were positive only by PCR but were negative by microscopy. Microscopically positive, but PCR negative trypanosomatids were identified only to the family level (Trypanosomatida) and were not included in Table 2. Overall, 96.4% (n = 107) of trypanosomatids were identified to the genus or species levels, and obtained sequences were deposited at the GenBank (PV918715, PV918756–PV918760, PV918766, PV918876, PV918892–PV918894, PV918921–PV918925). The morphometrical data on the microscopically investigated *Trypanosoma* trypomastigotes (the infective stage) from the guts of *Culicoides* are provided in Table 3.

**Figure 7:**
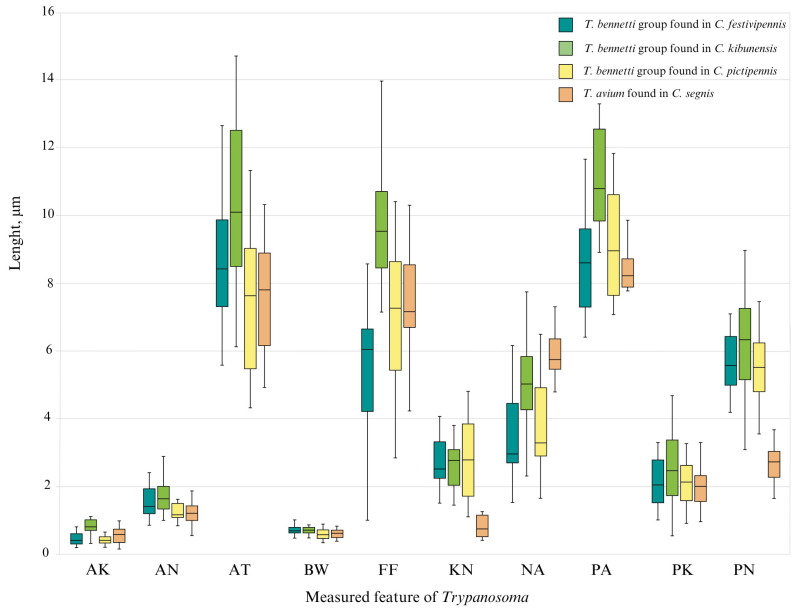
Morphometrical parameters of the *Trypanosoma bennetti* group trypanosomes in three different *Culicoides* species (*C. festivipennis*, *C. kibunensis*, and *C. pictipennis*) and *T. avium* found in *C. segnis*. Abbreviations are the same as in Table 3.

Morphometrical comparison of *T. bennetti* group trypanosomes from different biting midge species showed differences in their measurements (Figure 7). One-way ANOVA was used to assess the differences in morphometric traits between *T. avium* and *T. bennetti* group as well as *T. bennetti* group parasites detected in different *Culicoides* species. When comparing the morphometric traits of *T. bennetti* group in different biting midge species, statistically significant differences (*p* < 0.05) were found for all measured traits, except for the posterior end to kinetoplast (PK). In contrast, comparisons between two *Trypanosoma* species revealed statistically significant differences for the area of nucleus (AN), distance from kinetoplast to center of nucleus (KN), distance from center of nucleus to anterior end (NA), total length of the cell without free flagellum (PA), and distance from the posterior end to center of nucleus (PN), while the remaining traits did not differ significantly.

Tukey’s HSD post hoc test (*p* < 0.05) showed that *T. bennetti* in *C. kibunensis* stood out with a significantly different area of kinetoplast (AK) compared with this value in other *Culicoides* species. The area of nucleus (AN) of *Trypanosoma* in *C. kibunensis* was significantly larger than in *C. pictipennis* and *C. segnis*. *Trypanosoma bennetti* in *C. pictipennis* had significantly lower widths of cell through the center of nucleus (BW) than those both in *C. festivipennis* and *C. kibunensis*. The length of free flagellum (FF) was significantly longer in *C. kibunensis* compared with *C. festivipennis*, *C. pictipennis*, and *T. avium* in *C. segnis*. Additionally, the same parameter was significantly longer in *C. segnis* than in *C. festivipennis*. The distance from the center of nucleus to anterior end (NA) of *T. avium* in *C. segnis* was longer than for the *T. bennetti* group trypanosomes in *C. festivipennis* and *C. pictipennis*. *Culicoides kibunensis* also exhibited significantly larger values than *C. festivipennis* and *C. pictipennis. Trypanosoma avium* had a significantly shorter distance from the kinetoplast to center of nucleus (KN) and distance from the posterior end to kinetoplast (PK) compared with *T. bennetti* (Figure 7). No other statistically significant differences were detected.

### 3.4. Seasonality of Trypanosomatid Infection

A continuous investigation into *Culicoides* and the prevalence of trypanosomatids in them showed an increasing infection rate during the summer months: in June, the overall prevalence was 5.3% (n = 28) (n = 12, 3.6% in 2022 and n = 16, 6.9% in 2023); in July, it rose to 8.8% (n = 37) (8.8% during both years (n = 14 in 2022 and n = 23 in 2023)) and reached 11.2% (n = 32) in August (n = 9, 8.3% in 2022 and n = 23, 14.1% in 2023). The prevalence in May was 3.1% (n = 9) (0% in 2022 and n = 9, 6.1% in 2023) and in September, it was 1.4% (n = 1) (n = 1, 2.7% in 2022 and 0% in 2023). The calculated differences of the trypanosomatid prevalences between different months were statistically significant (*p* < 0.0001).

**Figure 8:**
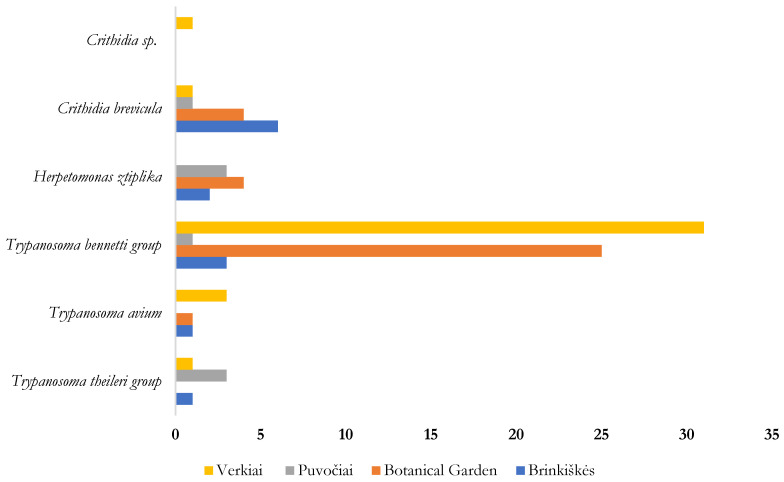
The number of biting midges infected with trypanosomatids at different study sites in the period 2022–2023.

The highest prevalence of avian trypanosomes (*T. bennetti* group trypanosomes and *T. avium*) was recorded in Verkiai and the Botanical Garden, while most of the mammalian infected trypanosomes (*T. theileri* group) were found in insects collected in Brinkiškės and Puvočiai (Figure 8).

## 4. Discussion

We investigated both monoxenous and dixenous trypanosomatids in *Culicoides* biting midges in order to evaluate how the prevalence of these parasites in insects changed through the warm season (from May to September) in the eastern Baltic region. Research on the impact of environmental changes to the spread of disease agents is not new [1], but despite this, information on the impact of meteorological factors on protozoans, which cause disease to vertebrates that have no economic importance (such as wild mammals and birds), is much less.

The first report on *Culicoides* seasonality in Lithuania was published in 2006 [49], which covered the data from 2000 to 2003 and recorded 21 species of *Culicoides* with the highest abundance in the first part of August. The second report on *Culicoides* seasonal surveillance in Lithuania was published in 2021 [21], with data obtained during 2016–2019; the findings of this study included records of 22 *Culicoides* species, and the highest diversity and abundance reported in June. Overall, 34 species of *Culicoides* have been recorded in Lithuania [50,51,52]. Most of the studies on biting midges carried out in Lithuania have concentrated on avian blood parasites, mostly hemosporidian [53,54,55,56] and *Trypanosoma* [5,57].

During this study, the highest numbers of insects were captured in June: as the season progressed, the numbers of biting midges decreased with each month. June can be characterized not only by the highest number of specimens collected, but also by the highest number of species collected (Figure 2), which confirms the previously published data [55]. The most abundant species was *C. kibunensis*: adults were caught from June to the first part of September (Figure 4), with the highest abundance detected in July (38.7%). An investigation from Spain also showed that the *C. kibunensis* peak is in July [58]. *Culicoides pictipennis* was the most abundant in May (66%). The same as in previous studies [21,59], our results confirm that *C. pictipennis* is an early species whose flying activity is the highest in late spring and decreases as the season progresses. The activity of *C. festivipennis* increased in the second part of summer, confirming the findings of a previous study that showed that *C. festivipennis* captures peaked in late autumn in Sassari, Italy [60]. *Culicoides obsoletus* group species are the most widespread in Europe [61,62,63,64], which start to fly in early spring (when the mean land surface temperature exceeds 10 °C) and ends in late autumn (when the mean land surface temperature becomes lower than 4 °C) [65]. Our study showed *C. obsoletus* group activity from early May until late September, although it was different during the two years of study: in 2022, these insects were active from June to September, and in 2023, they were captured from May to June (Figure 4). During our investigation, the peak in *C. obsoletus* group activity was in June (19.5%) and decreased throughout the season, although in September, this species group accounted for the majority of collected biting midges (90%). The abundance of *C. obsoletus* group insects was negatively related to the air temperature (Figure 5), and these insects were also collected continuously throughout the whole warm season in previous studies [21].

Our results obtained using RDA (Figure 5) can be related to known information on biting midge preferences for their breeding sites. The abundance of *C. segnis* was positively related to temperature and precipitation, although previous studies found that these insects prefer non-flooded and short-term flooded freshwater wetland habitats [66]. Precipitation becomes one of the main elements of such habitat existence—as there is more rainfall, more suitable habitats are available for these insects to develop. Similarly, *C. kibunensis* was found to prefer soil habitats located in stagnant water bodies, swamps, and eutrophic freshwater bodies [67,68,69] for which precipitation is a very important factor, as precipitation contributes to the recharge of groundwater, which in turn supports small water bodies [70] and swamps [71]. *Culicoides festivipennis*, which was found to be very flexible in its tolerance for the range of environmental conditions [67], and *C. pallidicornis*, which prefers acidic soil (3.6–5.0 pH) with low levels of moisture [72], were also positively related to temperature. Interestingly, studies have shown that *C. kibunensis* and *C. pallidicornis* prefer the same sites for breeding and might be present alongside each other [72,73,74]. *Culicoides festivipennis* was more abundant in August of 2023; in that year, August was the warmest summer month with an average day temperature of 19.1 °C. Both *C. punctatus* and *C. obsoletus* group biting midges have very diverse preferences for breeding environments, moreover, in addition to open wild habitats, they often prefer the dung of different animals [17,75]. In such cases, precipitation and wind become less important, especially if breeding sites are inside closed farms and animal sheds. In the 2021 report on the changes in Lithuanian climate [76], a comparison of the previously used SCN (standard climate norm) (1981–2010) and the current SCN (1991–2020) showed that the annual air temperature in Lithuania has increased by 0.5 °C, the annual precipitation has slightly decreased, and the average annual wind speed has decreased by 0.2 m/s. Compared with other dipteran vector groups, *Culicoides* biting midges are poor fliers and have low self-propelled flight, which makes their appetitive behavior strongly dependent on environmental parameters such as temperature, humidity, light intensity, and wind speed (>3 ms)^−1^ [20,77].

To date, *Culicoides* are well-known vectors of avian blood trypanosomes [4,5,57]. *Trypanosoma avium* is well-studied in both vertebrate hosts and vectors: it was the first trypanosomatid described from avian blood and later found to be infecting raptor birds [78] where Simuliidae black flies were described as vectors of this parasite [79], while *T. bennetti* Kirkpatrick et al., 1986 and *T. everetti* Molyneux, 1973 were described later (from the American kestrel *Falco sparverius* in 1986 and from the black-rumped waxbill *Estrilda t. troglodytes* in 1973) [78,80], where both were later found to be infecting passerine birds in the wild [5,81,82], however, information on their vectors is still scarce. The possibility of *Trypanosoma* development in *Culicoides* guts was suggested 64 years ago [83], and few *Culicoides* species have been proven to be *Trypanosoma* vectors through experimental research [4,5]. Nevertheless, wild biting midge infection research is less common, and without experimental confirmation, it is impossible to claim that the infected biting midge species are vectors of trypanosomatids. Our current study supports the results of previous research [57] that *T. bennetti* group trypanosomes can often be found in *C. pictipennis* and *C. kibunensis* biting midges, although to confirm their vector status further, experimental research, preferably, is needed. The morphometrical analysis conducted in this study comparing the *T. bennetti* group trypomastigotes found in three different biting midge species (*C. festivipennis*, *C. kibunensis*, *C. pictipennis*) showed statistically significant morphometrical differences within the *T. bennetti* group trypanosomes for all of the measured traits, except for posterior end to kinetoplast (PK) (Table 3, Figure 7). The *Trypanosoma bennetti* group consists of at least two species, *T. everetti* and *T. bennetti*, which can be characterized by low (0.4–1.3%) genetic differences in partial SSU rDNA sequences [5] and currently, morphometric data are only available for *T. bennetti* hematozoic trypomastigotes in experimentally infected *Culicoides nubeculosus* (Meigen, 1830) biting midges [4]. Comparing morphometrical data from this study to the previously published data of *T. bennetti* measurements in *C. nubeculosus,* it can be stated that the same *Trypanosoma* species trypomastigotes morphometrically differ in different *Culicoides* species (Figure 7). For morphological result confirmation, PCR-based analysis of the partial SSU rDNA gene was conducted, however, using this molecular marker, it is impossible to separate *T. bennetti* from *T. everetti*, and we can only speculate that these morphometrical differences could be the result of different *Trypanosoma* species present in insect guts. Morphometrical data in this case are crucial for a better understanding of the differences between *T. everetti* and *T. bennetti* species in insect hosts, although it must be complemented with PCR-based diagnosis and the morphology of *Trypanosoma* from bird blood for more accurate results.

It is important to mention that *Trypanosoma avium* was found in wild *C. segnis* several times [5,57], and it seems that this insect species might be a vector of the parasite. However, as it is very complicated to collect the target species in the wild and to establish a colony of *C. segnis* in the laboratory, the vector status remains unproven, and more investigation is needed to answer this question.

During our study, we found two species of monoxenous trypanosomatids: *Herpetomonas ztiplika* and *Crithidia brevicula*. *H. ztiplika* is a regular for *Culicoides*, although being described from *C. kibunensis*, it was also found in other species of the *C. obsoletus* group, *C. pictipennis*, and *C. punctatus* during this study, and more evidence of this parasite in *C. obsoletus* insects has been shown in a previous study [5]. As for *Crithidia brevicula*, which was described from blood-sucking mosquitoes [84,85], our study only confirmed previous findings [57] that *Culicoides* are also hosts of this parasite. Insects do not require blood meals from vertebrates to become infected with these parasites as they can become infected by feeding on infected prey, fresh feces (i.e., coprophagy), necrophagy, cannibalism, or contaminated substrates (which is common for species that breed and/or feed on moist substrates such as decaying organic matter, wetlands, manure, etc.) [23,86]. However, it is possible for vertebrates to become infected with monoxenous parasites if a previously infected insect feeds on the vertebrates’ blood [87,88]. The highest diversity of monoxenous insects was found in two insect orders—Diptera and Hymenoptera [23]—and even a few monoxenous species, such as *Sergeia podlipaevi* Svobodová et al., 2007 [89] and two species of *Herpetomonas* (*H. trimorpha* Zídkova et al., 2010 [90], and *H. ztiplika* [91]), were described directly from *Culicoides* biting midges.

We observed a variation in the prevalence of mammalian and avian trypanosomes across different study sites: more biting midges infected with mammalian trypanosomes were found in Brinkiškės and Puvočiai, while more insects infected with avian trypanosomes were found in Verkiai and the Botanical Garden (Figure 8). As previously mentioned, trapping sites in Brinkiškės and Puvočiai were located further from the city, closer to natural mixed temperate forests (Brinkiškės), and rural areas (Puvočiai), where more ruminants are present (cattle in Puvočiai and possible wild cervids in Brinkiškės), while trapping locations in the Botanical Garden and Verkiai were located closer to the city, where birds are more commonly present than large mammals. Additionally, our findings provide information on the feeding preferences of *Culicoides*: *C. kibunensis* and *C. obsoletus* group biting midges showed both mammalophilic and ornithophilic feeding behaviors, as both avian and mammalian trypanosomes were found in these insects. Notably, the feeding preferences of these insects were evidenced not only by the undigested blood found in the examined engorged females, but also by the parasites detected in parous females [53,92,93].

We found that trypanosomatid infection in *Culicoides* biting midges increased during the summer months: while 5.3% of biting midges were infected with trypanosomatids in June, the prevalence increased in July and in August (Figure 2). Similar research conducted on hemosporidian parasites showed that although the highest abundance of *Culicoides* was detected in June, July had the highest prevalence of biting midges infected with hemosporidian parasites, which indicates that it could be the best time for hemosporidian parasite research in wild-caught vectors, as July might be the month with the most active hemosporidian parasite transmission [55]. The same conclusion can be made based on our results: August is the most suitable month for the investigation of dixenous trypanosomatids, and it may be that during this time of the season, the most active transmission of these parasites occurs in the wild.

Only 54.0% of the biting midges investigated were positive for trypanosomes using both PCR and microscopy, and the rest were positive only by one of the approaches (42.4% by PCR and 3.6% by microscopy). Similar results have been found in Bolivia, where the PCR prevalence of *T. cruzi* in the feces of *Triatoma infestans* Klug, 1834 was 81%, while microscopy detected only 56% [94]; later, the efficacy of two PCR protocols with microscopy on the same parasite in *T. infestans* was compared, and it was found that while microscopy detected only 9.4% of infections, PCR showed estimates that were almost double (16.9–18.4% depending on the PCR method) [95]. Although studies have indicated that PCR might be more sensitive than microscopy, it may fail due to low parasitemia in the host [33,35], while microscopy has been shown to be as reliable a tool as PCR-diagnostics and is necessary for morphological species confirmation and for certain parasites, even species-level identification [33,96]. A combination of methods might provide more accurate results regarding species and developmental stage identification in insect hosts.

## 5. Conclusions

The results of this study show that the highest *Culicoides* abundance was recorded in June, while the highest prevalence of trypanosomatids in biting midges was recorded in August in the eastern Baltic region, which allows us to conclude that August might be the most suitable month for dixenous trypanosomatid investigation in this region, as it might be that during this time of the season, the most active transmission of these parasites occurs in the wild. Moreover, the air temperature presided as the meteorological gradient structuring *Culicoides* species composition: *C. festivipennis*, *C. pallidicornis*, and *C. achrayi* were determined to be more associated with higher temperatures, while the abundance of *C. segnis* and *C. kibunensis* seemed to be influenced by both air temperature and precipitation, although temperature took the lead; for *C. impunctatus*, *C. obsoletus* group, and *C. pictipennis*, they appeared to be weakly or negatively associated with the investigated meteorological variables. Although *Culicoides* is known for avian trypanosome transmission, our results note that mammalian trypanosomes from the *T. theileri* group can also be found in these insects, although further research is needed to better understand the development of these parasites and *Culicoides* vectorial capacity.

Morphometrical comparison of two *Trypanosoma* species from the gut of *Culicoides* revealed statistically significant differences for some traits, but the *T. bennetti* group trypanosomes from different biting midge species also showed differences in their measurements. Our results indicate that the combination of morphometrical analysis together with PCR-based methods is crucial for accurate parasite identification and a better understanding of host–parasite interactions.

## Figures and Tables

**Table 1:** Primers used in this study.

Primer Name	Sequence 5′–3′	Expected Amplicon Size	References
LCO 1490 (forward)	GGTCAACAAATCATAAAGATATTGG	~600 bp	Folmer et al., 1994 [46]
HCO 2198 (reverse)	TAAACTTCAGGGTGACCAAAAAATCA	~600 bp	
TRYP 763 (forward)	CATATGCTTGTTCAAGGAC	~1000 bp	Sehgal et al., 2001 [45], Valkiūnas et al., 2011 [43]
TRYP 1016 (reverse)	CCCCATAATCTCCAATGGAC	~1000 bp	
TRYP 99 (forward)	TCAATCAGACGTAATCTGCC	~700 bp	
TRYP 957 (reverse)	CTGCTCCTTTGTTATCCCAT	~700 bp	

**Table 2:** Trypanosomatids in the investigated *Culicoides* females collected in 2022–2023 (based on PCR).

	May	June	July	August	September	Total no. of Parasites (n Dixenous/n Monoxenous)
*Culicoides obsoletus *group	*Herpetomonas ztiplika* (1)	*H. ztiplika* (1) *T. theileri* group (1)	*T. bennetti* group (1)	*H. ztiplika* (2)	*H. ztiplika* (1)	2/5
*C. pictipennis*	*Trypanosoma bennetti* group (8)	*H. ztiplika* (2) *T. bennetti *group (7) *Crithidia brevicula* (1)	*H. ztiplika* (1) *C. brevicula* (2)			15/6
*C. impunctatus*		*C. brevicula* (1) *T. theileri *group (1)	*C. brevicula* (4)			1/5
*C. segnis*		*T. avium* (2)	*T. avium* (5) *Trypanosoma* sp. (1)			8/3
			*C. brevicula* (3)			
*C. punctatus*		*H. ztiplika* (2) *T. theileri *group (1)				1/2
*C. kibunensis*		*T. bennetti *group (5) *T. theileri *group (1) *T. avium* (1)	*T. bennetti *group (13) *T. theileri *group (1)	*T. bennetti *group (23) *T. theileri *group (1) *Trypanosoma* sp. (2)		47/0
*C. newsteadi*			*C. brevicula* (1)			0/1
*C. festivipennis*		*T. bennetti *group (1)	*Trypanosoma* sp. (1) *T. bennetti *group (2)	*T. bennetti *group (4)		8/0
*C. achrayi*			*C. brevicula* (1)			0/1
*C. grisescens*			*C. brevicula* (1)			0/1
*Culicoides* sp.		*C. brevicula* (1)				0/1

**Table 3:** Morphometric parameters (µm) of the metacyclic trypomastigotes of the *T. bennetti* group in the guts of three *Culicoides* species (*C. festivipennis*, *C. kibunensis*, and *C. pictipennis*) and *T. avium* in *C. segnis*. Minimum and maximum values are provided, followed in parentheses by the mean and SD.

Feature	Measurements (µm)			
	*C. festivipennis*	*C. kibunensis*	*C. pictipennis*	*C. segnis*
	*T. bennetti* gr. (n = 18)	*T. bennetti* gr. (n = 20)	*T. bennetti* gr. (n = 18)	*T. avium* (n = 17)
AK	0.2–0.9 (0.5 ± 0.2)	0.3–1.7 (0.9 ± 0.4)	0.2–0.8 (0.4 ± 0.1)	0.2–1.4 (0.6 ± 0.3)
AN	0.9–2.4 (1.5 ± 0.4)	1.0–2.9 (1.7 ± 0.5)	0.8–2.8 (1.3 ± 0.4)	0.6–2.7 (1.3 ± 0.5)
AT	5.6–12.6 (8.6 ± 1.8)	6.1–14.7 (10.3 ± 2.6)	4.3–11.3 (7.4 ± 2.1)	4.9–10.3 (7.6 ± 1.6)
BW	0.5–1.1 (0.7 ± 0.2)	0.5–1.1 (0.7 ± 0.1)	0.4–0.9 (0.6 ± 0.2)	0.4–0.8 (0.6 ± 0.1)
FF	1.0–8.6 (5.5 ± 1.9)	7.1–14.0 (9.8 ± 1.7)	2.8–10.4 (6.8 ± 2.1)	4.2–10.3 (7.5 ± 1.5)
KN	1.5–4.1 (2.7 ± 0.7)	1.5–3.8 (2.6 ± 0.6)	1.1–4.8 (3.0 ± 1.2)	0.5–2.4 (0.9 ± 0.5)
NA	1.5–6.2 (3.5 ± 1.3)	2.3–7.7 (5.0 ± 1.3)	1.7–6.5 (3.6 ± 1.3)	3.6–7.3 (5.8 ± 0.9)
PA	6.4–13.7 (8.8 ± 2.0)	8.9–13.3 (11.1 ± 1.4)	7.1–11.8 (9.3 ± 1.7)	6.5–9.9 (8.3 ± 0.8)
PK	1.0–3.3 (2.1 ± 0.7)	0.5–4.7 (2.6 ± 1.2)	0.9–3.3 (2.1 ± 0.7)	1.0–4.7 (2.2 ± 0.9)
PN	2.5–7.1 (5.5 ± 1.1)	3.1–14.9 (6.6 ± 2.4)	3.6–7.5 (5.6 ± 1.1)	1.7–3.7 (2.7 ± 0.5)
AN/AT	0.1–0.3 (0.2 ± 0.04)	0.1–0.3 (0.2 ± 0.1)	0.1–0.3 (0.2 ± 0.06)	0.1–0.3 (0.2 ± 0.1)
BW/PA	0.04–0.2 (0.09 ± 0.03)	0.05–0.1 (0.1 ± 0.01)	0.04–0.1 (0.06 ± 0.01)	0.05–0.1 (0.1 ± 0.01)
PK/PA	0.1–0.5 (0.3 ± 0.1)	0.1–0.5 (0.2 ± 0.1)	0.1–0.5 (0.2 ± 0.1)	0.1–0.6 (0.3 ± 0.1)
PN/KN	1.1–3.4 (2.1 ± 0.6)	1.6–7.5 (2.7 ± 1.4)	1.4–4.5 (2.1 ± 0.8)	1.1–6.4 (3.6 ± 1.6)
PN/NA	0.6–3.3 (1.8 ± 0.7)	0.6–3.5 (1.4 ± 0.7)	0.6–3.7 (1.7 ± 0.7)	0.3–0.8 (0.5 ± 0.1)
PN/PA	0.3–0.8 (0.6 ± 0.1)	0.3–1.4 (0.6 ± 0.2)	0.3–0.8 (0.6 ± 0.1)	0.2–0.4 (0.3 ± 0.1)

AK—area of kinetoplast; AN—area of nucleus; AT—area of trypomastigote; BW—width of body through center of nucleus; FF—length of free flagellum; KN—kinetoplast to center of nucleus; NA—center of nucleus to anterior end; PA—total length without free flagellum; PK—posterior end to kinetoplast; PN—posterior end to center of nucleus; BW/PA—body width index; PK/PA, PN/NA, PN/PA—nuclear index; PN/KN—kinetoplast index.

## Data Availability

All data generated during this study are included in this published article and are publicly available in the GenBank and Mendeley data repositories https://data.mendeley.com/datasets/wpyp8nbs59/1.
